# Neutral Buoyancy as a Simple Approach to Simulated Microgravity

**DOI:** 10.1007/s13770-025-00781-2

**Published:** 2026-01-13

**Authors:** Ho Yong Kim, Sungwook Kang, Se Heang Oh

**Affiliations:** 1 Department of Nanobiomedical Science, Dankook University, Cheonan, 31116 Republic of Korea; 2 Department of Smart Ocean Mobility Engineering, Changwon National University, Changwon, 51140 Republic of Korea; 3 Department of Biomedical Sciences & Biosystems, Dankook University, Cheonan, 31116 Republic of Korea

**Keywords:** Microgravity, Neutral buoyancy, Differentiation

## Abstract

**Background:**

It is well recognized that interesting biological phenomena occur in various organisms in microgravity. However, real microgravity research is limited by cost and accessibility. Furthermore, current ground-based microgravity simulators often cause shear stress and vibration, which restrict the accurate reproduction of a real microgravity environment. This study aimed to develop a simple, low-cost, and reproducible simulated microgravity system based on neutral buoyancy to reproduce an environment similar to that of real space.

**Methods:**

A neutral buoyancy medium (NBM) was created by adjusting the density of conventional cell culture medium through mixing with density gradient medium (Ficoll-Paque™, Percoll™, and Optiprep™). The buoyancy stability of human bone marrow-derived mesenchymal stem cell (*h*BMSC) spheroids was examined experimentally and by computational fluid dynamics (CFD). The effects of neutral buoyancy-based simulated microgravity (3D-sim-μg) on *h*BMSC stemness and trilineage differentiation (osteogenic, adipogenic, and chondrogenic) were compared with normal gravity.

**Results:**

Optiprep-based NBM (Optiprep™/cell culture medium, 20/80 v/v) maintained a stable suspension of *h*BMSC spheroids for 14 days. CFD analysis confirmed near-zero static pressure under neutral buoyancy, reproducing a microgravity-like environment. *h*BMSC spheroids in 3D-sim-μg showed enhanced expression of pluripotency markers and suppressed osteogenic differentiation, with increased adipogenic and chondrogenic expression compared to normal gravity.

**Conclusion:**

The neutral buoyancy-based system effectively simulates key microgravity-associated cellular behaviors, including maintenance of stemness and lineage-specific differentiation. This approach provides a simple and accessible platform for various microgravity research endeavors.

**Supplementary Information:**

The online version contains supplementary material available at 10.1007/s13770-025-00781-2.

## Introduction

Just as the quest to find a new water route to Asia inspired Christopher Columbus’s voyages, humanity's aspirations and curiosity about space propelled the development of spaceflight. Through spaceflight, researchers have discovered that various organisms, including humans/animals, plants, and bacteria, exhibit unique biological phenomena in the microgravity environment of space that differ from those on Earth. These include the activation of genes regulating cellular pathways [1], epigenetic fine-tuning of specific genes [2], alteration in metabolic homeostasis [1], immune dysregulation [3, 4], cytoskeletal rearrangement [5], changes in adhesion molecules [5–7], hemoglobin gene switching [8], oxidative damage in mitochondria [9], telomeric length alteration [10], abnormal plant growth due to auxin rearrangement in roots [11], altered antibiotic susceptibility in bacteria [12], and accelerated proliferation of non-motile bacteria [13]. These biological insights gained in microgravity have prompted new demands and opportunities in biomedical fields [14]. Various research teams are conducting groundbreaking research on the International Space Station (ISS) to uncover new and unforeseen biological insights in microgravity, though the research opportunities are somewhat limited due to significant financial constraints, transportation challenges, and infrequent flight opportunities [15]. To address these challenges, systems such as parabolic flights, drop towers (for real microgravity) [16, 17]; and 2D/3D clinostats, rotating wall vessel (RWV) bioreactors, and random positioning machines (RPMs) (for simulated microgravity) have been developed to create microgravity conditions on Earth [17, 18]. Simulated microgravity is achieved in 2D/3D clinostats and RWV by free-floating in a fluid subject to rotational forces, and in RPM by randomly rotating two axes to cancel the gravity vector. Additional methods of simulating microgravity include the use of magnetic or acoustic standing waves to levitate cells within a medium [19–21]. Several research teams have noted that many biological phenomena observed in these simulated microgravity systems parallel those noted on the ISS [15]. For instance, the osteogenic differentiation of human mesenchymal stem cells (*h*MSCs) is inhibited in both simulated microgravity systems and microgravity aboard the ISS, while pathways related to adipogenesis and chondrogenesis are promoted [22–24]. Additionally, various stem cells tend to maintain their stemness rather than differentiate into specific cell types in both types of microgravity environments [25–31]. However, cells in simulated microgravity systems on the ground are often exposed to various types of external forces depending on the underlying mechanism of each platform. In systems such as RWV bioreactors and RPMs, cells are subjected to fluid dynamic effects, including shear stresses from rotational movement, collisions within the culture vessel, vibrations, and centrifugal acceleration [16, 32, 33]. In contrast, magnetic levitation-based systems can impose forces resulting from the magnetic field itself, which interacts with the intrinsic magnetic susceptibility of cells or the medium [16, 21]. Similarly, acoustic levitation platforms utilize standing acoustic waves to counteract gravity and levitate cells, introducing localized pressure nodes and oscillatory forces that may affect cell behavior [20, 21]. These factors can lead to inconsistent outcomes and low reproducibility, making it difficult to determine if the results are solely due to simulated microgravity or a combination of multiple external factors [16, 32, 33]. Furthermore, the duration of microgravity in parabolic flights and drop towers is typically very short, lasting only a few seconds [15, 34]. Therefore, to achieve reliable real and simulated microgravity, it is imperative to develop systems that minimize the impact of external factors and provide prolonged periods of microgravity.

In 1840, physicist Joseph Plateau proposed the "Plateau Principle”, which demonstrates that oil droplets achieve a perfect spherical shape when their density is equal to that of a surrounding mixture of water and alcohol (buoyancy counteracts gravity) [35]. Lebert and Häder reported that the addition of 5% Ficoll™, a densifying agent, to the medium results in the impaired graviperception of *Euglena gracilis* [36]. The National Aeronautics and Space Administration (NASA) astronauts trained in a buoyant underwater environment to simulate spacewalks conventionally [37]. Based on these concepts, we hypothesized that using a medium with a density similar to that of cell matrices would allow simulating a microgravity environment in space through neutral buoyancy, where the net force resulting from the buoyancy (F_*B*_) and gravitational forces (F_*g*_) approaches zero. We speculated that this neutral buoyancy system could imitate microgravity more effectively and simply than previous methods, without the constraints of time and space/equipment [37–39]. In this study, we developed a cell culture medium capable of controlling neutral buoyancy by mixing cell culture medium (CCM) with commonly used density gradient medium (DGM; Ficoll-Paque™, Percoll™, and Optiprep™). Initially, we tested whether this neutral buoyancy medium (NBM) could replicate the well-known cell behaviors (e.g., maintenance of stemness [26, 27], suppression of osteogenic differentiation, and enhancement of chondrogenic/adipogenic differentiation [22, 23]) in microgravity using human bone marrow-derived mesenchymal stem cell (*h*BMSC) spheroids, thereby proposing NBM as a novel platform for microgravity research.

## Methods and methods

### Cell culture and cell spheroids formation

The *h*BMSCs were procured from StemCell Technologies and cultured in Dulbecco’s Modified Eagle Medium (DMEM) containing 20% Fetal Bovine Serum (FBS), and 1% penicillin–streptomycin (CCM) [40]. The *h*BMSCs, at a concentration of 3 × 10^5^ cells/mL, were seeded into ultra-low attachment (ULA) 96-well plates (Thermo Fisher Scientific) in 100 μL volumes and incubated overnight in a standard incubator for spheroid formation.

### Fabrication of neutral buoyancy medium (NBM)

Optimal NBM composition was determined by preparing a DGM-CCM mixture at various concentrations matching the spheroid density. Added concentrations were 20, 40, 60, 80, and 100% for Ficoll-Paque™ (Ficoll-Paque™/CCM; 20/80, 40/60, 60/40, 80/20, and 100/0 [v/v]); 5, 10, 15, 25, and 30% for Percoll™ (Percoll™/CCM; 5/95, 10/90, 15/85, 25/75, and 30/70 [v/v]); and 5, 10, 15, 25, and 30% for Optiprep™ (Optiprep™/CCM; 5/95, 10/90, 15/85, 25/75, and 30/70 [v/v]). The density of the mixed medium was quantified using the Eq. (1):

1 $${\rho }_{medium}=({\rho }_{1}{V}_{1}+{\rho }_{2}{V}_{2})/({V}_{1}+{V}_{2})$$

where *ρ*_*medium*_ (g/mL) represents the density of the mixture medium, *ρ*_*1*_ represents the density of CCM, *V*_*1*_ (mL) represents the volume of CCM, *ρ*_*2*_ represents the density of each DGM, and *V*_*2*_ represents the volume of each DGM.

The behavior of cell spheroids in the NBM was observed to assess which could maintain neutral buoyancy.

### Height change and morphology of *h*BMSC spheroids in NBM

Cell experiments were conducted to evaluate the duration of neutral buoyancy for cell spheroids in the selectively chosen NBMs [*Percoll* (Percoll™/CCM, 30/70 (v/v)) and *Optiprep* (Optiprep™/CCM, 20/80 (v/v))] and to examine how their morphology changes with exposure time to neutral buoyancy. A well (ULA 96-well plate) containing a cell spheroid was filled with 100 μL of each NBM and incubated for 14 days. The height changes of the suspended cell spheroids in each well were measured to assess the duration of neutral buoyancy. The size of cell spheroids incubated in NBMs or CCM at 14 days of cell culture was observed via a light microscope (Eclipse Ts2R; Nikon). The cell culture medium was exchanged every two days.

### Cytotoxicity

To assess the cytotoxicity of NBMs, *h*BMSCs (in CCM) were seeded in 6-well culture plates (Corning; cell density, 3 × 10^4^ cells/well) and incubated for 24 h to facilitate cell adhesion. Subsequently, the cells were treated with *Percoll* and *Optiprep* and further incubated for 7 days. The cytotoxicity of each NBM was determined using Cell Counting Kit-8 (CCK-8; Dojindo).

### Computational fluid dynamics analysis (CFD) model

The macroscopic particle model (MPM) in Fluent 2022 R2 (ANSYS) was utilized to analyze the behavior of cell spheroids in varying densities of cell culture medium. MPM is a computational method used for simulating the behavior of large particles in fluid flows. For MPM simulations, cell spheroids with a diameter of 500 μm and a density of 1.08 g/mL were employed. The total simulation time was 5 s, and the transient simulation was executed. For CFD simulations, a test well was modeled with a diameter of 6.35 mm and a height of 7.5 mm, consistent with the dimensions of a ULA 96-well plate. The total number of elements was 25,346, and the number of nodes was 110,301 (Supplement Fig. 1). The medium within the test tube was assumed to have densities of 1.02 g/mL (CCM), 1.08 g/mL (*Optiprep*), 1.13 g/mL (30% Optiprep™-based medium), and a viscosity of 1.050 mPa·s.

### Stemness maintenance and trilineage differentiation of *h*BMSCs

To determine whether *Optiprep*, which can provide neutral buoyancy for cell spheroids to simulate certain aspects of microgravity, a well (ULA 96-well plate) containing a cell spheroid was filled with 100 μL of *Optiprep* and incubated for 7 days. The cell behaviors of *h*BMSC spheroids in *Optiprep* (3D-sim-μg) were compared with those of *h*BMSC spheroids (3D-1 g) and monolayer *h*BMSC (2D-1 g) under normal gravity (1 g) conditions in CCM. To promote differentiation of *h*BMSCs into specific cells, a cell culture medium with the following composition was used: the Optiprep™ and osteogenic medium (CCM with 0.1 μM dexamethasone, 50 μg/mL ascorbic acid 2-phosphate, 10 mM β-glycerophosphate, 2 mM glutamine, 1 mM sodium pyruvate) [Optiprep™/osteogenic medium, 20/80 (v/v)]; the Optiprep™ and adipogenic medium (CCM with 1 μM dexamethasone, 0.2 mM indomethacin, 0.5 mM 3-isobutyl-1-methylxanthine, 1 mM sodium pyruvate, 5 μg/mL insulin) [Optiprep™/adipogenic medium, 20/80 (v/v)]; and the Optiprep™ and chondrogenic medium [CCM with 0.1 μM dexamethasone, 50 μg/mL ascorbic acid 2-phosphate, 1 mM sodium pyruvate, 40 μg/mL proline, a 1:100 v/v ratio of ITS, and 10 ng/mL transforming growth factor-β1 (TGF-β1; R&D systems)] [Optiprep™/chondorgenic medium, 20/80 (v/v)]. Except for TGF-β1, all reagents were sourced from Sigma-Aldrich.

### Quantitative RT-PCR

To compare the expression of target genes, total RNA from cell spheroids in each group (2D-1 g, 3D-1 g, and 3D-sim-μg) was extracted using NucleoZOL (Macherey–Nagel). This RNA was converted to complementary DNA (cDNA) using PrimeScript RT Master Mix (TAKARA). Subsequently, the target genes were amplified using TaqMan primers and Universal PCR Master Mix (Thermo Fisher Scientific), following amplification cycles of 95°C for 1 min, 60 °C for 30 s, and 72 °C for 1 min. The specific TaqMan Primers (Thermo Fisher Scientific) used were: OCT4 (Hs04260367_gH), SOX2 (Hs04234836_s1), NANOG (Hs02387400_g1), RUNX2 (Hs00231692_ml), osteocalcin (OCN; Hs00609452_gl), PPARγ (Hs01115513_m1), CEBP/α (Hs00269972_s1), SOX9 (Hs00165814_m1), aggrecan (ACN; Hs00153936_m1), and glyceraldehyde 3-phosphate dehydrogenase (GAPDH; Hs02786624_g1), with GAPDH serving as the housekeeping gene.

### Immunoblotting

To compare the expression of the target proteins, cell spheroids from each group (2D-1 g, 3D-1 g, and 3D-sim-μg) were lysed using a 1X Protease Inhibitor Cocktail containing radioimmunoprecipitation assay (RIPA) buffer (GenDEPOT). The proteins were separated using sodium dodecyl sulfate–polyacrylamide gel electrophoresis (SDS-PAGE) and transferred to a polyvinylidene difluoride (PVDF) membrane (Millipore) with a 0.45 μm pore size. The blots were blocked with EzBlock Chemi (ATTO Corporation) for 1 h and then incubated with primary antibodies for OCT4 (2750S), SOX2 (3579S), NANOG (4903S) (Abclonal; 1:1,000 dilution), and GAPDH (5174S) (Cell Signaling Technology; 1:1,000 dilution) at 4 °C for 12 h with shaking. The blots were then washed with Tris-buffered saline with 0.1% Tween-20 (TBS-T) and incubated with horseradish peroxidase-conjugated secondary antibodies (Thermo Fisher Scientific; 1:2,000 dilution) for 1 h. Detection was conducted using the ImageQuant LAS 4000 imager (GE Healthcare) after developing the blots with an enhanced chemiluminescence (ECL) solution (GenDEPOT).

### Immunofluorescence staining

To visualize the expression of target markers, cell spheroids were fixed in 4% paraformaldehyde for 20 min at 4 ^◦^C, and specimens embedded in optimal cutting temperature (OCT) compound were sliced into 6-μm-thick sections. The sections were permeabilized with a 1% Triton X-100 solution, blocked with 10% bovine serum albumin (in TBS containing 1% BSA), and incubated with primary antibodies [RUNX2 (ab192256), OCN (ab198228), PPARγ (ab133612), CEBP/α (ab40761), SOX9 (ab185966), and CAN (ab3778), (Abcam; each 1:100 dilution except CEBP/α at 1:50 dilution)] for 12 h at 4 ^◦^C. This was followed by incubation with secondary antibodies [Alexa Fluor 488 conjugated goat anti-rabbit IgG and Alexa Fluor 647 conjugated goat anti-mouse IgG (Abcam; both 1:200 dilution)] for 1 h at room temperature. Nuclei were stained with DAPI (VectorLab), and immunofluorescence images were captured using a confocal laser microscope (LSM 700; Carl Zeiss). Subsequent staining was performed to compare differentiation behaviors in each group.

#### Histological analysis

6-μm-thick cell spheroid slices (center of cell spheroid) were stained for 15 min with 2% alizarin red S (ARS) in distilled water for osteogenic differentiation, 10 min with 0.5% oil red O (ORO) in isopropanol for adipogenic differentiation, and 20 min with 1% alcian blue (AB) in 3% acetic acid for chondrogenic differentiation, respectively. The stained sections were examined with an optical microscope (Eclipse Ts2R; Nikon). For histological staining quantification, ARS-stained slices were dissolved in 100 μL of 10% acetic acid and shaken for 30 min. Absorbance was measured at 405 nm using a spectrophotometer (Spark10M; TECAN). ORO was extracted from the ORO-stained slices using 100 μL of isopropanol and its absorbance was determined at 492 nm. AB-stained slices were destained with 100 μL of 6 M guanidine-HCl for 30 min and absorbance was measured at 620 nm. The unstained cell spheroid slices were used as background for the absorbance of each extraction solution.

#### Statistics

Each experiment was conducted independently at least three times, and results were expressed as mean ± standard deviation. Statistical analyses were performed using GraphPad Prism software (GraphPad Software Inc.). The student’s t-test and one-way analysis of variance (One-way ANOVA), followed by a post-hoc Tukey’s test, were utilized. The significance level was set at *p* < 0.05.

## Results and discussion

### Establishment and optimization of a neutral buoyancy-based simulated microgravity system

**Fig. 1:**
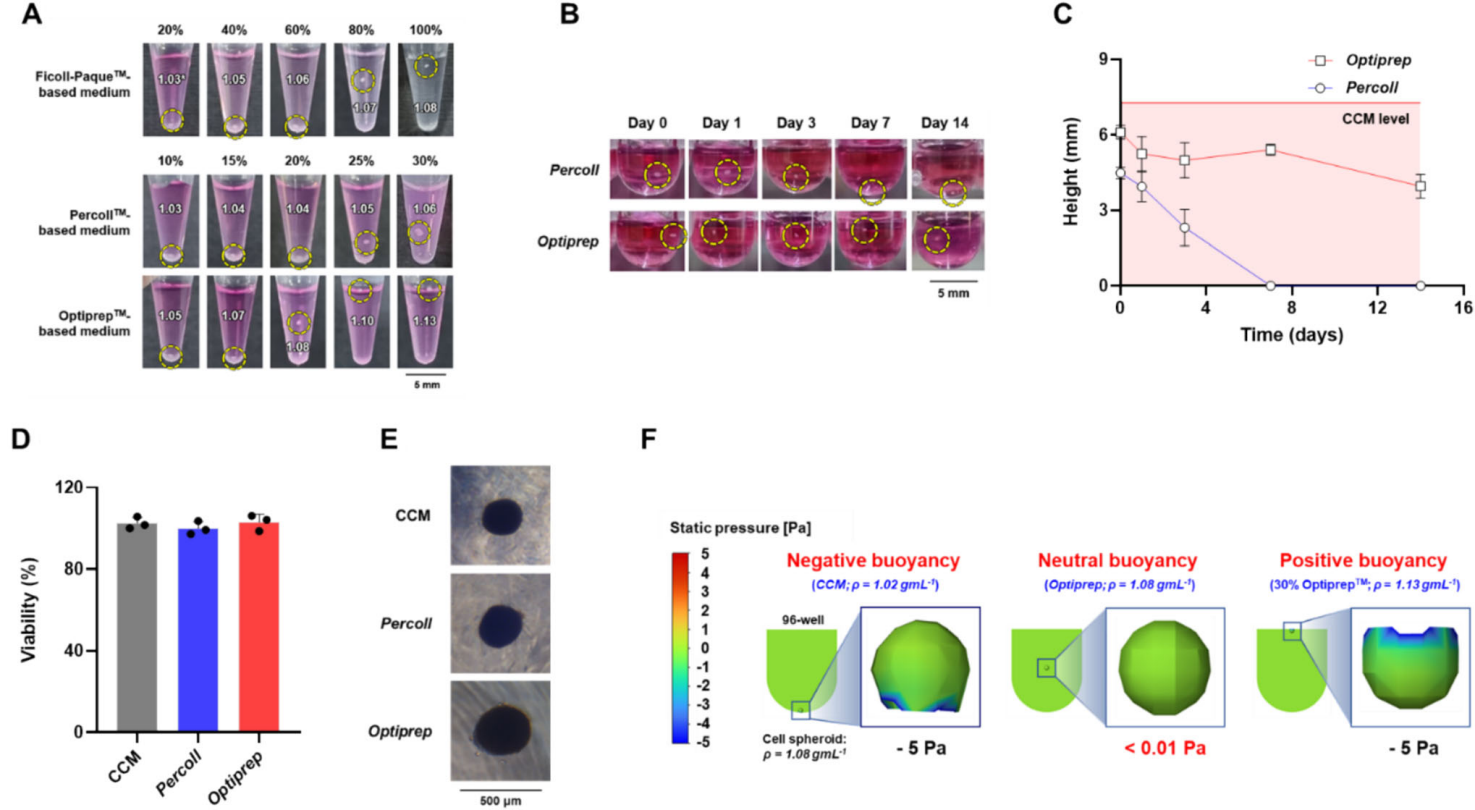
Characterization of neutral buoyancy cell culture medium (NBM) for microgravity simulation. **A** Suspension of *h*BMSC spheroids in NBM containing various density gradient medium (DGM; Ficoll-Paque™, Percoll™, and Optiprep™) compositions (^*^, medium density), **B** and **C** images and changes in flotation height of cell spheroids over time in *Percoll* and *Optiprep*, **D** and **E** viability and morphology of cell spheroids in *Percoll* and *Optiprep* (after 7 days of cell culture) (n = 3). **F** Computational fluid dynamics (CFD) analysis of cell spheroids in medium under negative, neutral, and positive buoyancy conditions

Three DGMs (Ficoll-Paque™, Percoll™, and Optiprep™) were used to determine the optimal neutral buoyancy for *h*BMSC spheroids by adjusting the cell culture medium density. It was found that spheroids could be suspended in mediums of varying densities, achieving neutral buoyancy (Fig. 1A). Based on each medium’s density [CCM (Dulbecco’s Modified Eagle Medium [DMEM] containing 20% Fetal Bovine Serum [FBS]), 1.023 g/mL [41]; Ficoll-Paque™, 1.077 g/mL [42]; Percoll™, 1.13 g/mL [43]; and Optiprep™, 1.32 g/mL [44]], concentrations of 30% Percoll™ (*Percoll*; density, 1.06 g/mL), 80% Ficoll-Paque™ (*Ficoll*; density, 1.07 g/mL), and 20% Optiprep™ (*Optiprep*; density, 1.08 g/mL) (in CCM) were initially chosen based on their similarity in density range (1.06 to 1.08 g/mL) to that of *h*BMSC spheroids, which facilitates neutral buoyancy. However, the mixture with just 20% CCM in *Ficoll* was discounted from further experiments due to its insufficiency of vital components required for cell viability.

The ability to maintain neutral buoyancy over a sufficient period for cell culture was assessed by monitoring the height changes of cell spheroids suspended in *Percoll* and *Optiprep*. Cell spheroids in *Percoll* initially suspended at heights ranging from 4.27 to 4.74 mm (CCM height, 7.45 mm) gradually sank over 7 days until they reached the bottom, whereas cell spheroids in *Optiprep* remained suspended for 14 days with heights varying from 6.11 to 3.95 mm, demonstrating a neutral buoyancy environment (Fig. 1B, C). The sedimentation of cell spheroids in *Percoll* over time can be attributed to the endocytosis of silica nanoparticles (size, 15–30 nm) from the colloidal Percoll™ and the consequent gradual increase in the density of the cell spheroids [43, 45, 46]. In contrast, it has been reported that Optiprep™, consisting of a non-ionic and iso-osmolar molecule named iodixanol, is confined to the extracellular space [47]. Consequently, *Optiprep* is less likely to be internalized by cells, allowing cell spheroids to remain stably suspended in a neutral buoyancy environment of the NBM without density changes. Mita et al. also reported that the using the Optiprep™ gradient medium to purify cells can improve cell (β-cell) survival and increase the success of organ (islet) transplantation. [48].

To determine the cytotoxicity of *Percoll* or *Optiprep*, cell viability in each NBM was compared with that in cells cultured in normal CCM. It was observed that there were no significant differences in cell viability among the groups, indicating an absence of substantial cytotoxicity (Fig. 1D). Interestingly, a progressive increase in cell spheroid size was noted, correlating with the duration of exposure to neutral buoyancy (cell spheroid size, *Optiprep* > *Percoll* > CCM) (Fig. 1E). This phenomenon can be attributed to the compression of cell spheroids by gravity, leading to a denser cellular arrangement [49].

A computational fluid dynamics (CFD) analysis was conducted for the mathematical estimation of fluids and forces to compare the magnitude of the external force exerted on the cell spheroids under neutral (gravity = buoyancy), positive (gravity < buoyancy), and negative (gravity > buoyancy) buoyancy conditions (Fig. 1F and Supplement video 1). Setting the cell spheroid (size, 500 μm) density at 1.08 g/mL and the medium density at 1.02 g/mL (density of CCM; negative buoyancy) resulted in the cell spheroid gradually sinking to the bottom, with a calculated static pressure of—5 Pa (A negative value means that an external pressure is being applied to the cell spheroid). Conversely, setting the medium density at 1.13 g/mL (density of 30% Optiprep™-based medium; positive buoyancy) caused the cell spheroid to slowly rise and contact the surface, with a static pressure of—5 Pa. Thus, an imbalance of gravitational and buoyant forces can serve as an external force on the cell spheroid. However, with the medium density set to 1.08 g/mL (density of *Optiprep*; neutral buoyancy), the cell spheroids were suspended in the medium, confirming previous observations where the static pressure on the cell spheroid was virtually zero (< 0.01 Pa). This almost negligible static pressure creates a condition of floating, emulating the microgravity seen in space [50]. Collectively, these findings demonstrate that a cell culture medium providing neutral buoyancy can effectively simulate microgravity on Earth, offering a viable platform for studying various biological phenomena linked to microgravity.

### Neutral buoyancy-based simulated microgravity reproduces space microgravity-associated stemness and differentiation behaviors of *h*BMSC spheroids

**Fig. 2:**
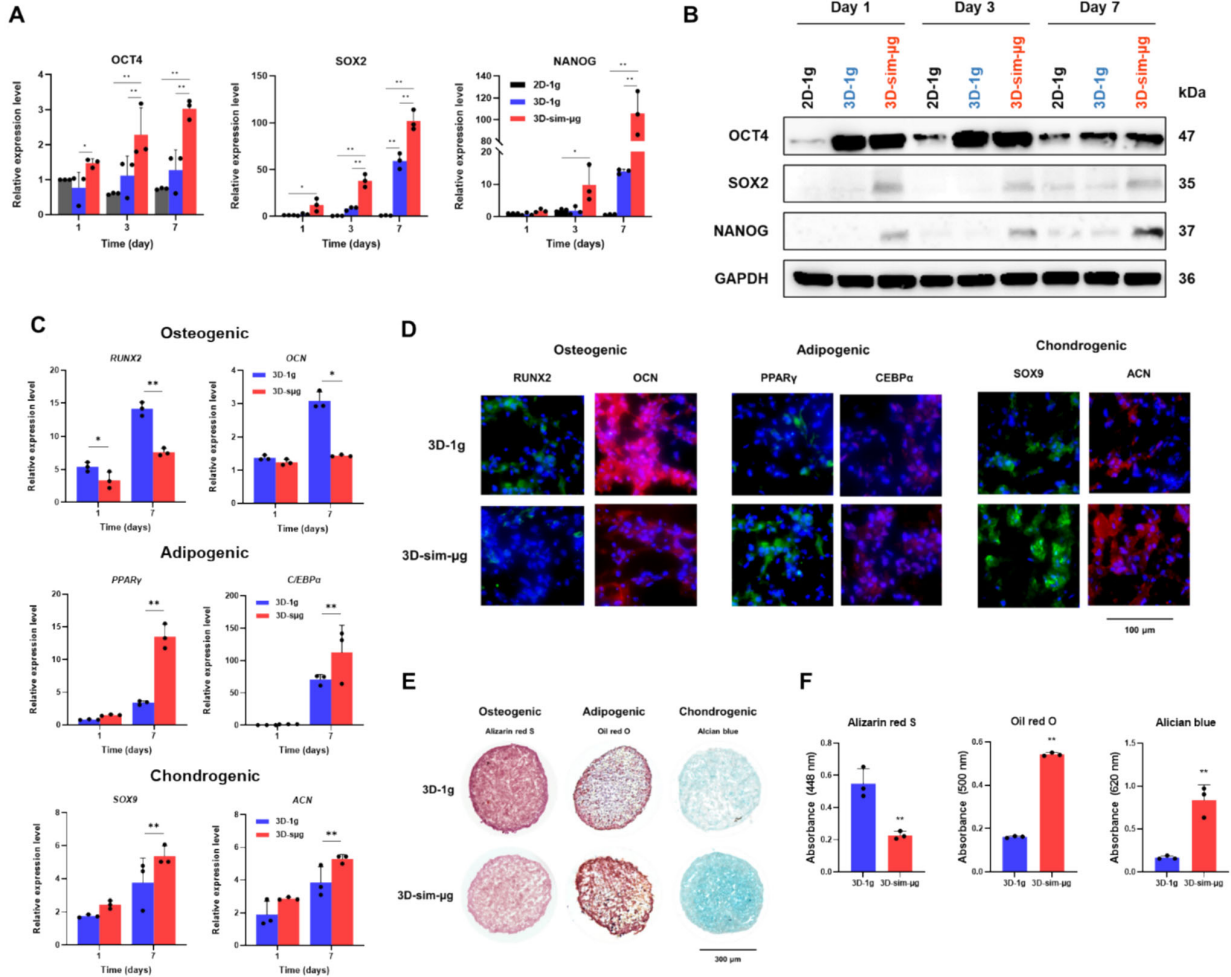
The feasibility of the simulated microgravity in *Optiprep* with a neutral buoyancy regarding the stemness maintenance and trilineage differentiation of *h*BMSCs was evaluated. **A** qRT-PCR analysis and **B** western blotting determined the expression of OCT4, SOX2, and NANOG (stemness markers) in *h*BMSCs cultured at 3D-sim-μg (in *Optiprep*), 3D-1 g (in CCM), and 2D-1 g (in CCM). **C** qRT-PCR analysis, **D** immunofluorescence staining, **E** histological analysis, and **F** quantitative analysis of osteogenic differentiation markers (RUNX2, OCN, and alizarin red S), adipogenic differentiation markers (PPARγ, CEBP/α, and Oil red O), and chondrogenic differentiation markers (SOX9, ACN, and Alcian blue) of *h*BMSCs cultured at 3D-sim-μg and 3D-1 g (n = 3; *p < 0.05 and **p < 0.01)

Cell experiments were conducted to assess whether exposure to *Optiprep,* providing simulated microgravity conditions (sim-μg), induces stemness-associated cellular behaviors, similar to those observed in mesenchymal stem cells under microgravity [26–28, 51]. Behaviors of three-dimensional (3D) *h*BMSC spheroids in sim-μg (3D-sim-μg) were compared to 3D *h*BMSC spheroids (3D-1 g) and two-dimensional (2D) monolayers of *h*BMSC (2D-1 g) cultured under normal gravity (1 g) in CCM. It was observed that the key genes (Fig. 2A) and proteins (Fig. 2B and Supplement Fig. 2) related to stem cell pluripotency, such as OCT4, SOX2, and NANOG, were significantly upregulated in 3D-μg compared to other groups, indicating better maintenance of stemness in *h*BMSCs. It was also confirmed that cell spheroids with a 3D structure, which more closely mirrors the in vivo environment in terms of cell–cell and cell-extracellular matrix interactions, provide a superior environment for maintaining stemness compared to monolayer cells [52, 53]. These findings imply that enhanced Wnt signaling, a crucial pathway regulating stem cell stemness, via a synergistic effect of the 3D culture system and simulated microgravity, is a plausible mechanism. It has been noted that MSCs cultured in 3D enhance the Wnt signaling pathway by reducing cytoskeletal tension [54, 55]. Additionally, it is reported that Wnt signaling is further enhanced in real and simulated microgravity due to the reduction of cytoskeletal tension [56–58].

It is well known that microgravity in space affects stem cell differentiation by suppressing osteogenic differentiation and promoting chondrogenic/adipogenic differentiations [59, 60]. The differentiation behaviors of 3D *h*BMSC spheroids in *Optiprep* containing each differentiation factor (3D-sim-μg) into target cell types (bone, cartilage, and fat) were compared with those of 3D *h*BMSC spheroids in 1 g (3D-1 g) of CCM containing each differentiation factor. The osteogenic-related transcription factor (RUNX2) and ECM protein (osteocalcin, OCN) were downregulated in 3D-sim-μg compared with 3D-1 g at day 7 of cell culture. In contrast, significant upregulation of adipogenic transcription factors (PPARγ and CEBP/α), chondrogenic transcription factors (SOX9), and chondrogenic-related proteoglycan (aggrecan, ACN) was observed in 3D-sim-μg relative to 3D-1 g (Fig. 2C and 2D). Additionally, alizarin red S (ARS; a marker for osteogenic differentiation) was suppressed, while oil red O (ORO; a marker for adipogenic differentiation) and alcian blue (AB; a marker for chondrogenic differentiation) were enhanced under simulated microgravity (3D-sim-μg) compared to 1 g (3D-1 g) (Figs. 2E, F and Supplement Fig. 3). These results can be explained by signaling pathways involving YAP/TAZ and RUNX2. It is known that YAP/TAZ, which is activated in response to extracellular mechanical stimuli is poorly translocated to the nucleus in microgravity, thus acting as an inhibitor of nuclear transcription factors such as RUNX2 [61, 62]. A decrease in RUNX2 leads to an increase in SOX9, which has a transcriptional repressor function against RUNX2, resulting in a decrease in osteogenic differentiation and an increase in chondrogenic differentiation [63]. The reduction of RUNX2 also induces an increase in adipogenesis, activating the PPARγ and CEBP/α pathways, which are known to be inhibited by RUNX2 [58]. These findings demonstrate that neutral buoyancy for cell spheroids can be achieved by simple density control of the cell culture medium and that neutral buoyancy can be adapted to new simulated microgravity (mechanical unloading) systems, simulating the cell behaviors observed in real microgravity systems, including those on the ISS.

Although this study demonstrated that the NBM can provide simulated microgravity which can reflect some aspects of real microgravity, there are still questions about the long-term effects of the added Optiprep™ on the cells and whether organelles can also experience microgravity. These concerns are important issues that need to be thoroughly investigated and addressed in future follow-up studies.

## Conclusion

We developed a cell culture system that can provide neutral buoyancy for cell spheroids by mixing CCM and DGM (Ficoll-Paque™, Percoll™, and Optiprep™). This neutral buoyancy-based system induces cellular responses that resemble previously reported behaviors in mesenchymal stem cells exposed to real or simulated microgravity, such as enhanced maintenance of stemness, reduced osteogenic differentiation, and increased adipogenic/chondrogenic tendencies. These parallels suggest that the system may provide a practical and accessible platform for investigating specific aspects of cellular adaptation to microgravity. Furthermore, we expect that this neutral buoyancy-based system can be extended to various cell types or organoid systems, thus may be a useful tool to investigate cellular behaviors under microgravity conditions. We believe that this adaptability may extend to the fields of regenerative medicine and space biology.

## Supplementary Information

Below is the link to the electronic supplementary material.

Supplementary Material1 (DOCX 820 KB)

Supplementary Material2 (MP4 659 KB)

## Data Availability

The raw data supporting the conclusions of this article will be made available by the corresponding author upon reasonable request.
