# Supplementary material for: Neutral Buoyancy as a Simple Approach to Simulated Microgravity
Source: Tissue Eng Regen Med. 2026 Jan 13;23(2):199–208. doi: 10.1007/s13770-025-00781-2 (PMC12855663; doi:10.1007/s13770-025-00781-2)
Supplement: Supplementary file 1 — Supplementary Material1 (DOCX 820 KB) [file 13770_2025_781_MOESM1_ESM.docx]

Supplementary figures

Neutral buoyancy as a simple approach to simulated microgravity

**Ho Yong Kim^1^, Sungwook Kang^2^, and Se Heang Oh^1, 3*^**

^1^Department of Nanobiomedical Science, Dankook University, Cheonan, 31116, Republic of Korea

^2^Department of Smart Ocean Mobility Engineering, Changwon National University, Changwon, 51140, Republic of Korea

^3^Department of Biomedical Sciences & Biosystems, Dankook University, Cheonan, 31116, Republic of Korea

*Author to whom any correspondence should be addressed.

E-mail addresses: seheangoh@dankook.ac.kr


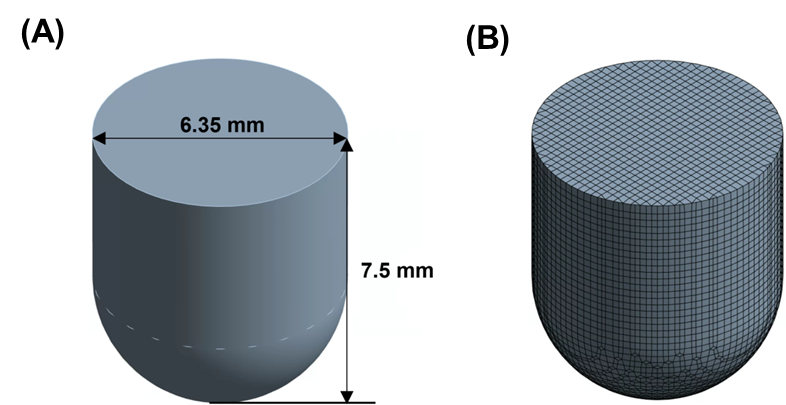


**Supplement Fig. 1**. 3-dimensional geometry (A) and total node model (B) for Computational fluid dynamics (CFD) analysis.


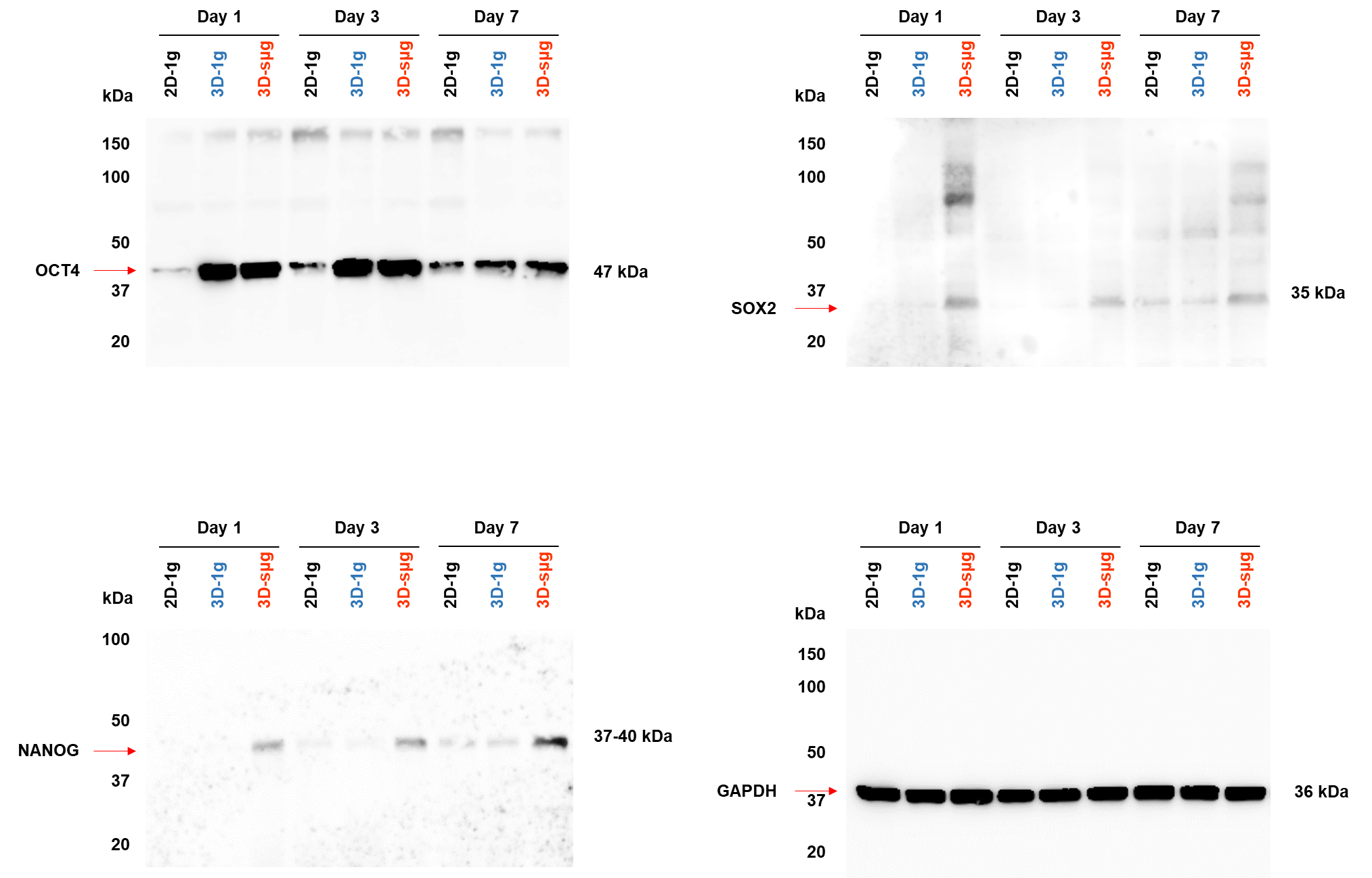


**Supplement Fig. 2**. The feasibility of the simulated microgravity in *Optiprep* with a neutral buoyancy regarding the stemness maintenance of *h*BMSCs was evaluated. Western blotting determined the expression of OCT4, SOX2, and NANOG (stemness markers) in *h*BMSCs cultured at 3D-sim-μg (in *Optiprep*), 3D-1g (in CCM), and 2D-1g (in CCM).


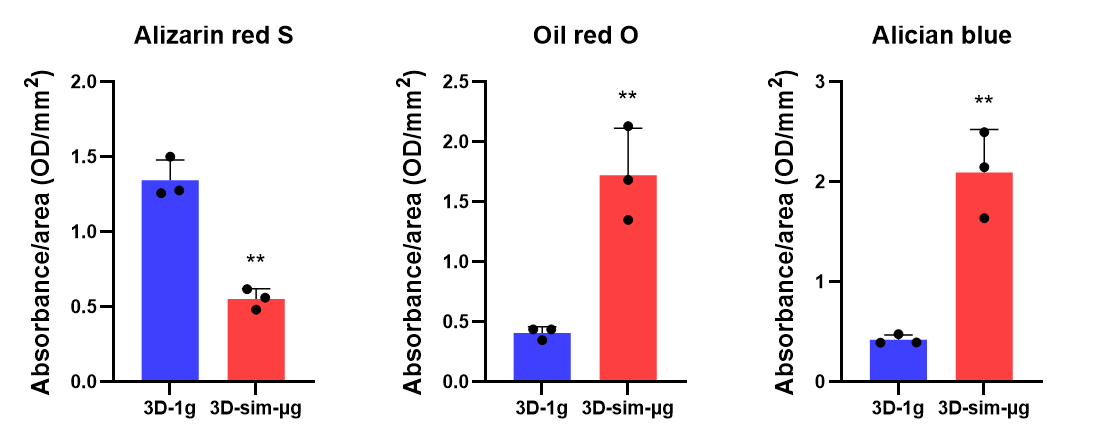


**Supplement Fig. 3.**  The feasibility of the simulated microgravity in *Optiprep* with a neutral buoyancy regarding the trilineage differentiation of *h*BMSCs was evaluated. Quantitative analysis [absorbance (OD)/section area (mm^2^)] of osteogenic differentiation marker (alizarin red S), adipogenic differentiation marker (Oil red O), and chondrogenic differentiation marker (Alcian blue) of *h*BMSCs cultured at 3D-sim-μg and 3D-1g (n = 3 and ***p* < 0.01).
